# Gender disparity in LDL-induced cardiovascular damage and the protective role of estrogens against electronegative LDL

**DOI:** 10.1186/1475-2840-13-64

**Published:** 2014-03-25

**Authors:** An-Sheng Lee, Wei-Yu Chen, Hua-Chen Chan, Jing-Fang Hsu, Ming-Yi Shen, Chia-Ming Chang, Henry Bair, Ming-Jai Su, Kuan-Cheng Chang, Chu-Huang Chen

**Affiliations:** 1 Department of Medicine, Mackay Medical College, New Taipei, Taiwan; 2 Division of Cardiology, China Medical University Hospital, Taichung City, Taiwan; 3 Graduate Institute of Clinical Medical Science, China Medical University, Taichung, Taiwan; 4 L5 Research Center, China Medical University Hospital, Taichung, Taiwan; 5 George R. Brown School of Engineering, Rice University, Houston, Texas, USA; 6 Graduate Institute of Pharmacology, National Taiwan University, Taipei, Taiwan; 7 Center for Lipid Biosciences and Department of Medicine, Kaohsiung Medical University, Kaohsiung, Taiwan; 8 Vascular and Medicinal Research, Texas Heart Institute, 6770 Bertner Avenue, MC 2-255, Houston, TX 77030, USA; 9 Department of Medicine, Baylor College of Medicine, Houston, Texas, USA

**Keywords:** Electronegative low-density lipoprotein, Metabolic syndrome, Cardiovascular disease, Aortic senescence, 17β-estradiol, Genistein

## Abstract

**Background:**

Increased levels of the most electronegative type of LDL, L5, have been observed in the plasma of patients with metabolic syndrome (MetS) and ST-segment elevation myocardial infarction and can induce endothelial dysfunction. Because men have a higher predisposition to developing coronary artery disease than do premenopausal women, we hypothesized that LDL electronegativity is increased in men and promotes endothelial damage.

**Methods:**

L5 levels were compared between middle-aged men and age-matched, premenopausal women with or without MetS. We further studied the effects of gender-influenced LDL electronegativity on aortic cellular senescence and DNA damage in leptin receptor–deficient (*db/db*) mice by using senescence-associated–β-galactosidase and γH2AX staining, respectively. We also studied the protective effects of 17β-estradiol and genistein against electronegative LDL–induced senescence in cultured bovine aortic endothelial cells (BAECs).

**Results:**

L5 levels were higher in MetS patients than in healthy subjects (*P <* 0.001), particularly in men (*P =* 0.001). LDL isolated from male *db/db* mice was more electronegative than that from male or female wild-type mice. In addition, LDL from male *db/db* mice contained abundantly more apolipoprotein CIII and induced more BAEC senescence than did female *db/db* or wild-type LDL. In the aortas of *db/db* mice but not wild-type mice, we observed cellular senescence and DNA damage, and the effect was more significant in male than in female *db/db* mice. Pretreatment with 17β-estradiol or genistein inhibited BAEC senescence induced by male or female *db/db* LDL and downregulated the expression of lectin-like oxidized LDL receptor-1 and tumor necrosis factor-alpha protein.

**Conclusion:**

The gender dichotomy of LDL-induced cardiovascular damage may underlie the increased propensity to coronary artery disease in men.

## Background

The accelerated development of atherosclerosis and premature aging of the cardiovascular system have been recognized in patients with metabolic syndrome (MetS) [1]; however, the underlying mechanisms remain unclear. Vascular aging is a feature of MetS that may represent the prodromal stage of atherosclerotic disease, favored by co-existing harmful stimuli such as dyslipidemia [2].

The potential role of electronegative low-density lipoprotein (LDL) in cardiovascular diseases is gaining a global recognition [3-9]. Increased proportions of electronegative LDL have been described in many conditions that define metabolic syndrome, such as hypertriglyceridemia [10], diabetes [4,11], insulin resistance [12], obesity [13], and hypertension [14]. Previously, we separated plasma LDL according to charge into 5 subfractions, L1-L5, by using anion-exchange chromatography and showed that plasma levels of L5—the most electronegative subfraction of LDL—are higher in patients with MetS and ST-segment elevation myocardial infarction (STEMI) than in healthy individuals [15]. L5 has a chemical composition unique from that of other LDL subfractions and has been shown to induce atherogenic processes [16,17], including endothelial cell apoptosis and mitochondrial dysfunction [18-20]. Although L5 has a role in promoting atherogenesis, it is not known whether L5 induces endothelial cell senescence, a pro-inflammatory and prothrombotic phenotype associated with arterial stiffness [21], endothelial dysfunction [22], and atherosclerosis [23]. Understanding whether L5 contributes to endothelial cell senescence may help to explain the accelerated development of atherosclerosis and the premature aging observed in MetS patients.

The risk of coronary artery disease (CAD) is greater in men than in premenopausal women [24], but the mechanisms are not completely understood. In addition, the frequency of cardiovascular disease rises in post-menopausal and in estrogen-deficient women [25]. Because LDL becomes more toxic as its electronegativity increases [16], we hypothesized that LDL electronegativity is higher in male patients with MetS than in female patients with MetS and may underlie the increased propensity to CAD observed in men. In addition, using mice with a proclivity to developing diabetes and atherosclerosis, we compared the effects of male and female LDL on cellular senescence and determined whether estrogen confers protection against the harmful effects of highly electronegative LDL.

## Materials and methods

### Human L5 study

LDL was isolated from the plasma of asymptomatic, middle-aged men and age-matched, premenopausal women who did (MetS patients, n = 30; 15 men and 15 women) or did not (control subjects, n = 26; 11 men and 15 women) meet 3 of the criteria for MetS according to the National Cholesterol Education Program-Adult Treatment Panel III guidelines [26]. Patients who were taking medications that may affect the electronegativity of LDL such as statins, angiotensin-converting enzyme inhibitors, and angiotensin receptor blockers were excluded [27]. All participants gave informed consent for the use of their plasma, and the study was conducted according to the principles in the Declaration of Helsinki. Patient characteristics, including waist circumference, systolic blood pressure, diastolic blood pressure, and levels of glucose, triglyceride, total cholesterol, high-density lipoprotein cholesterol (HDL-C), and LDL cholesterol (LDL-C) were determined according to standard operating procedures. LDL samples were further resolved into subfractions L1-L5 by using fast protein liquid chromatography (GE Healthcare, Pittsburgh, PA) with an anion-exchange UnoQ12 column (BioRad, Hercules, CA).

### Biochemical analysis of mouse blood plasma

All animal experiments were approved by the China Medical University Institutional Animal Care and Use Committee and were performed in accordance with the *Guide for the Care and Use of Laboratory Animals* published by the US National Institutes of Health (NIH Publication No. 85–23, revised 1996). Whole blood was drawn from mice by means of intubation via the common carotid artery, and plasma was obtained by using centrifugation. Using an automated Biochemical Analyzer (SP-4430, Spotchem EZ, Arkray), we examined plasma levels of glucose, total cholesterol, total triglyceride, HDL-C, and LDL-C. LDL (*d =* 1.030-1.063 g/mL) was isolated by using sequential ultracentrifugation as previously described [19]. Protein concentrations were determined by using the Lowry method [16]. The charge of LDL was analyzed by subjecting LDL samples to gel electrophoresis in 0.7% agarose, and the composition of delipidated LDL was analyzed by subjecting samples to SDS-PAGE. Relative mobility (Rf) was quantified as a measure of the distance migrated by a band divided by the distance migrated by the dye front. For Western blot analysis, proteins were transferred to PVDF membrane and probed by using antibodies against apolipoprotein CIII (ApoCIII) and ApoB100 (Academy Biomedical Company, Inc., Houston, TX). Images were acquired and analyzed by using the G-box imaging system (Syngene, Frederick, MD). To quantify the protein expression of ApoCIII, we normalized the band intensity with that of ApoB100 (internal control).

### Analysis of cellular senescence and DNA damage in mouse aortas

Male and female homozygous leptin receptor knock-out mice (*db/db*) and wild-type C57B6/J littermates (controls) were purchased from The Jackson Laboratory (Bar Harbor, ME) and were maintained on a 12-hour light/dark cycle with free access to food and water. After 6 months on a normal chow diet, all animals were anesthetized by means of 2% isoflurane inhalation, and the aorta of each mouse was removed for histologic examination.

For the detection of cellular senescence in the aortic endothelium, aortas were fixed for 10 minutes at room temperature in 2% formaldehyde/0.2% glutaraldehyde solution and were incubated at 37°C for 24 hours with fresh senescence-associated–β-galactosidase (SA-β-gal) staining solution (Cell Signaling Technology, Inc., Danvers, MA). Using images acquired with a Nikon D300s digital camera, we identified senescent cells marked by a blue color produced by the enzymatic reaction.

For the detection of DNA damage in the aortic endothelium, thoracic descending aortas were paraffin-embedded, and serial cross-sections were obtained from each sample. Slides were immunostained with anti–phospho-histone H2AX (anti-γH2AX) antibody (Cell Signaling Technology, Inc.) and analyzed by using an Olympus IX70 inverted microscope (Japan) with OPTRONICS digital microscope cameras (Goleta, CA).

To examine telomerase enzyme activity, aortas or subconfluent bovine aortic endothelial cells (BAECs) were homogenized in 3-[3-(cholamidopropyl) diethylammonio]-1-propane sulfonate (CHAPS) buffer. Homogenized tissues were then used to perform telomerase activity assays by using a modified telomeric repeat amplification protocol (TRAPeze RT Telomerase Detection Kit; Chemicon, Billerica, MA) according to the manufacturer’s instructions. Real-time PCR amplification and detection was performed with StepOnePlus Real-Time PCR Systems (Applied Biosystems, Grand Island, NY). Threshold cycle (Ct) measurements were obtained for determining telomerase activity. Values for Ct, which are inversely related to telomerase activity, were then adjusted with the standard curve by using the commercial TSR8 template.

### Analysis of cellular senescence in cultured BAECs

Subconfluent BAECs were incubated with a subapoptotic concentration (30 μg/mL) of LDL from male or female *db/db* or wild-type mice for 5 consecutive days. Cells were then either lysed in radio-immunoprecipitation assay lysis buffer (Sigma-Aldrich Co., St. Louis, MO) containing protease inhibitor cocktail and 0.1% Triton X-100 for Western blot analysis or were stained with SA-β-gal for the detection of senescent cells. A total of 500 cells were scored in 10 randomly selected fields to determine the percentage of SA-β-gal–positive cells.

To investigate the protective effects of estrogen against cell senescence induced by LDL from *db/db* mice, BAECs were pretreated with 17β-estradiol (10 nM; Biorbyt Ltd., UK) or its isoflavone analogue genistein (100 nM; LC Laboratories, MA), followed by exposure with 30 μg/mL LDL from male or female *db/db* or wild-type mice. For the detection of cell senescence, cells were stained with SA-β-gal as described above.

### Western blot analysis of lectin-like oxidized LDL receptor-1 (LOX-1) and tumor necrosis factor-α (TNF-α)

Using Western blot analysis, we examined the protein expression of LOX-1 (antibody from R&D Systems, Minneapolis, MN) and TNF-α (antibody from GeneTex Inc., Irvine, CA) in BAECs treated with 30 μg/mL LDL from male *db/db* mice or PBS in the presence or absence of pretreatment with 17β-estradiol (10 nM) or genistein (100 nM), as described previously [19].

## Results

### The increased percent of L5 in total LDL (L5%) in MetS patients is more pronounced in men than in women

**Figure 1:**
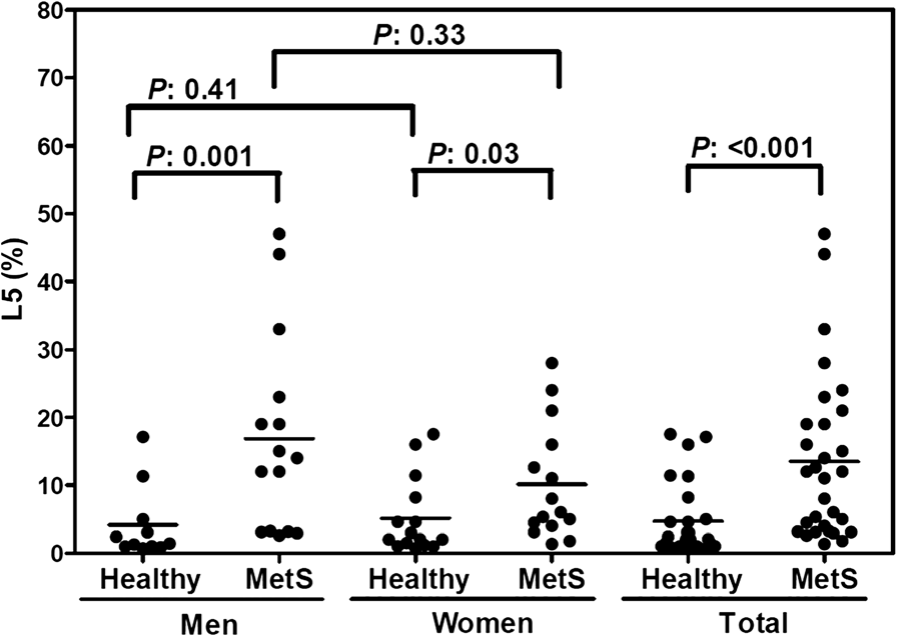
**Percent L5 in total LDL (L5%) among healthy control subjects and MetS patients, grouped by gender.** L5% was significantly higher in MetS patients than in healthy subjects. In addition, men with MetS had slightly higher L5% than did women with MetS. Values for L5% were determined by using fast protein liquid chromatography. The solid line indicates the mean value for each group.

We examined the L5% in MetS patients and healthy control subjects (Table 1 and Figure 1) and confirmed that L5% is significantly higher in MetS patients than in healthy subjects (*P <* 0.001). Although no significant difference was observed between men in total LDL-C, men with MetS had significantly higher L5% than did healthy men (*P =* 0.001). Furthermore, men with MetS had higher L5% than did premenopausal women with MetS, although the difference was not statistically significant (*P =* 0.33). Waist circumference was significantly different between control men and control women (Table 1; *P <* 0.05). No other significant differences were observed in characteristics between control men and control women or between MetS women and MetS men.

**Table 1:** Gender-stratified characteristics of MetS patients and healthy control subjects

	**Men**		**Women**		**Total**	
	**Control (n = 11)**	**MetS (n = 15)**	**Control (n = 15)**	**MetS (n = 15)**	**Control (n = 26)**	**MetS (n = 30)**
Age (years)	41.2 ± 9.8	48.9 ± 7.1*	49.3 ± 15.7	53.2 ± 4.4	45.9 ± 13.9	51.1 ± 6.2*
Waist (cm)	88.5 ± 10.6	106.4 ± 12.9**	74.4 ± 8.0^**#**^	106.2 ± 15.2**	80.4 ± 11.5	106.3 ± 13.9**
SBP (mmHg)	121.3 ± 9.6	136.2 ± 16.5*	120.1 ± 26.8	120.2 ± 14.8	120.6 ± 21.0	127.5 ± 17.3
DBP (mmHg)	81.5 ± 10.2	83.4 ± 9.8	73.0 ± 14.6	74.5 ± 9.6	76.6 ± 13.4	78.6 ± 10.5
Glucose (mg/dL)	89.2 ± 7.7	142.7 ± 59.1**	85.1 ± 7.4	102.4 ± 19.4**	86.9 ± 7.6	122.5 ± 47.8**
TC (mg/dL)	232.0 ± 47.8	196.9 ± 48.0	224.9 ± 60.0	220.0 ± 59.1	227.9 ± 54.2	208.5 ± 54.2
TG (mg/dL)	151.8 ± 95.0	180.9 ± 151.8	106.3 ± 95.5	144.1 ± 59.0*	125.6 ± 96.2	162.5 ± 114.7
HDL-C (mg/dL)	52.7 ± 15.8	44.0 ± 12.2	62.1 ± 13.7	54.0 ± 19.2	58.1 ± 15.1	49.0 ± 16.6*
LDL-C (mg/dL)	149.0 ± 37.9	120.9 ± 45.3	142.3 ± 52.1	143.9 ± 41.2	145.2 ± 45.9	132.8 ± 44.0
L5 (%)	4.1 ± 5.3	16.9 ± 14.6**	5.1 ± 5.6	10.1 ± 8.5*	4.7 ± 5.4	13.5 ± 12.2**

Values represent the mean ± standard deviation. *P*-values were determined by using the Wilcoxon rank sum test (Mann–Whitney U test). *MetS* metabolic syndrome; *SBP* systolic blood pressure; *DBP* diastolic blood pressure; *TC* total cholesterol; *TG* triglyceride; *HDL-C* HDL cholesterol; *LDL-C* LDL cholesterol. *P < 0.05, **P < 0.01 vs gender-matched control group; ^**#**^*P <* 0.05 vs male control group.

### Male *db/db* mice show increased DNA damage and aortic senescence

The *db/db* mouse is a well-established animal model for analyzing diabetic dyslipidemia and MetS, which precipitates obesity, insulin resistance, hyperglycemia, and leptin resistance [28]. We found that male and female *db/db* mice had higher body weights and plasma levels of fasting plasma glucose, total cholesterol, HDL-C, and LDL-C than did their gender-matched wild-type littermates after 6 months on a normal chow diet (Table 2). However, no significant differences were observed in these parameters between male and female *db/db* mice or between male and female wild-type mice.

**Table 2:** **Gender-stratified characteristics of leptin receptor–deficient (*db/db*) mice and wild-type (C57B6/J) littermates**

	**Male**		**Female**	
	**C57B6/J**^**# **^**(n = 8)**	***db/db***^***# ***^**(n = 8)**	**C57B6/J (n = 6)**	***db/db *** **(n = 5)**
Body weight (g)	36.50 ± 1.46	62.50 ± 3.33**	35.00 ± 0.84	57.80 ± 4.84**
Glucose (mg/dL)	206.88 ± 10.74	398.00 ± 19.89**	219.17 ± 9.04	307.20 ± 41.11*
TC (mg/dL)	74.63 ± 4.69	188.88 ± 16.00**	73.17 ± 2.23	183.60 ± 10.80**
TG (mg/dL)	47.13 ± 5.78	52.88 ± 6.24	41.33 ± 8.10	49.40 ± 7.05
HDL-C (mg/dL)	51.00 ± 3.29	97.25 ± 8.24**	50.83 ± 2.82	89.60 ± 7.92**
LDL-C (mg/dL)	14.20 ± 1.89	81.05 ± 11.29**	14.07 ± 4.08	84.12 ± 6.04**

Values represent the mean ± standard error of the mean. *TC* total cholesterol; *TG* triglyceride; *HDL-C* HDL cholesterol; *LDL-C* LDL cholesterol. Variation between groups was determined by using the two-independent-sample test with the Mann–Whitney U test for comparisons. *P < 0.05, **P < 0.01 vs gender-matched control group. ^#^No parameter was significantly different from that of female mice of the same genotype.

**Figure 2:**
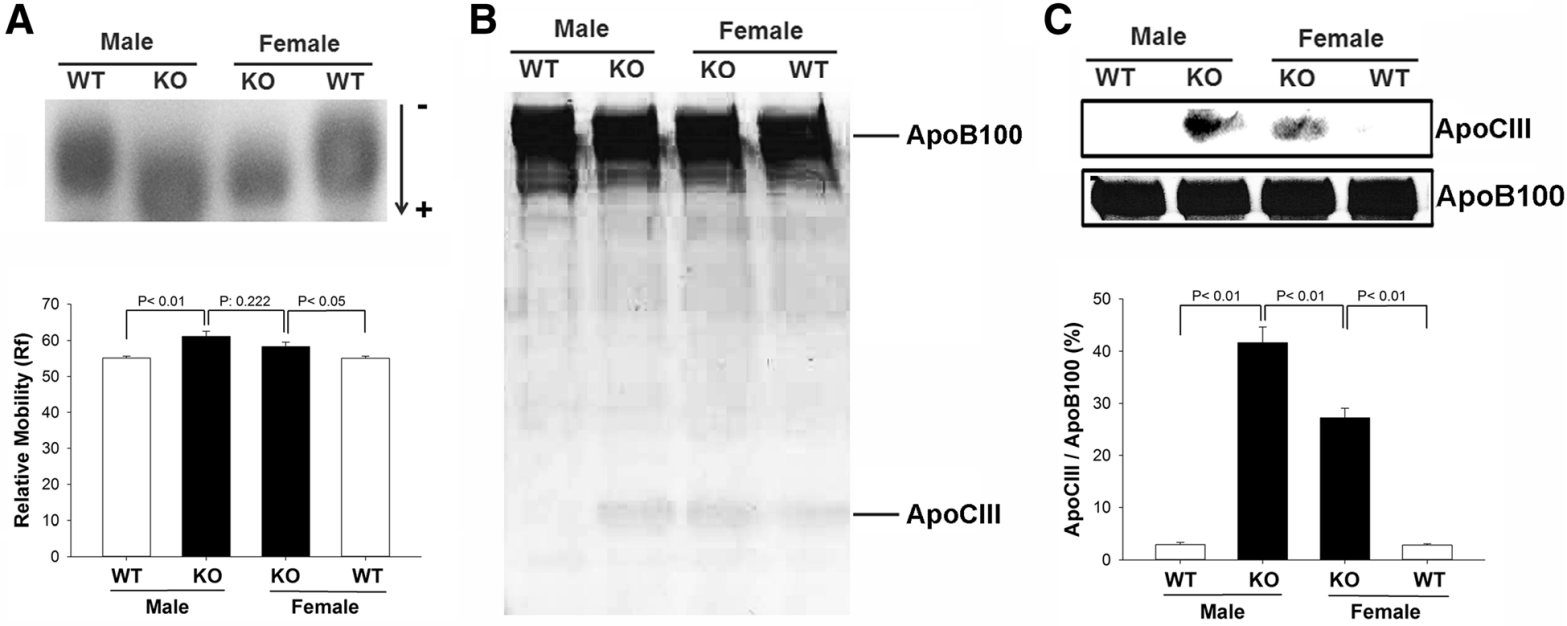
**Comparison of LDL electronegativity and protein composition between *db/db *and wild-type mice. A**, Representative results of agarose gel electrophoresis showing the relative electronegativity of LDL isolated from male and female *db/db* (KO) and wild-type (WT) mice. Relative mobility (Rf) was compared among groups. **B**, Representative results of SDS-PAGE showing the relative concentration of ApoCIII in LDL isolated from male and female *db/db* (KO) and wild-type (WT) mice. **C**, Western blot analysis of ApoCIII and ApoB100 protein levels in LDL isolated from male and female *db/db* (KO) and wild-type (WT) mice. The ratio of ApoCIII to ApoB100 was compared among groups. n = 5 for each group.

To compare the electronegativity of LDL among male and female *db/db* and wild-type mice, we analyzed LDL samples using agarose gel electrophoresis. In native agarose gel, LDL from male *db/db* mice migrated toward the anode faster than did LDL from male or female wild-type mice (Figure 2A), indicating that LDL from male *db/db* mice was more electronegative than LDL from wild-type mice. LDL from female *db/db* mice was only slightly more electronegative than LDL from male or female wild-type mice. No significant differences were observed in LDL electronegativity between males and females in either wild-type or *db/db* mice. When we examined the protein composition of LDL among male and female *db/db* and wild-type mice using SDS-PAGE, we observed a higher content of ApoCIII in *db/db* mice than in gender-matched wild-type mice (Figure 2B). These findings were confirmed by the results of Western blot analysis for ApoCIII (*P <* 0.01) (Figure 2C). In LDL from male *db/db* mice, ApoCIII levels were significantly higher than those in LDL from female *db/db* mice (*P <* 0.01) (Figure 2B and C).

**Figure 3:**
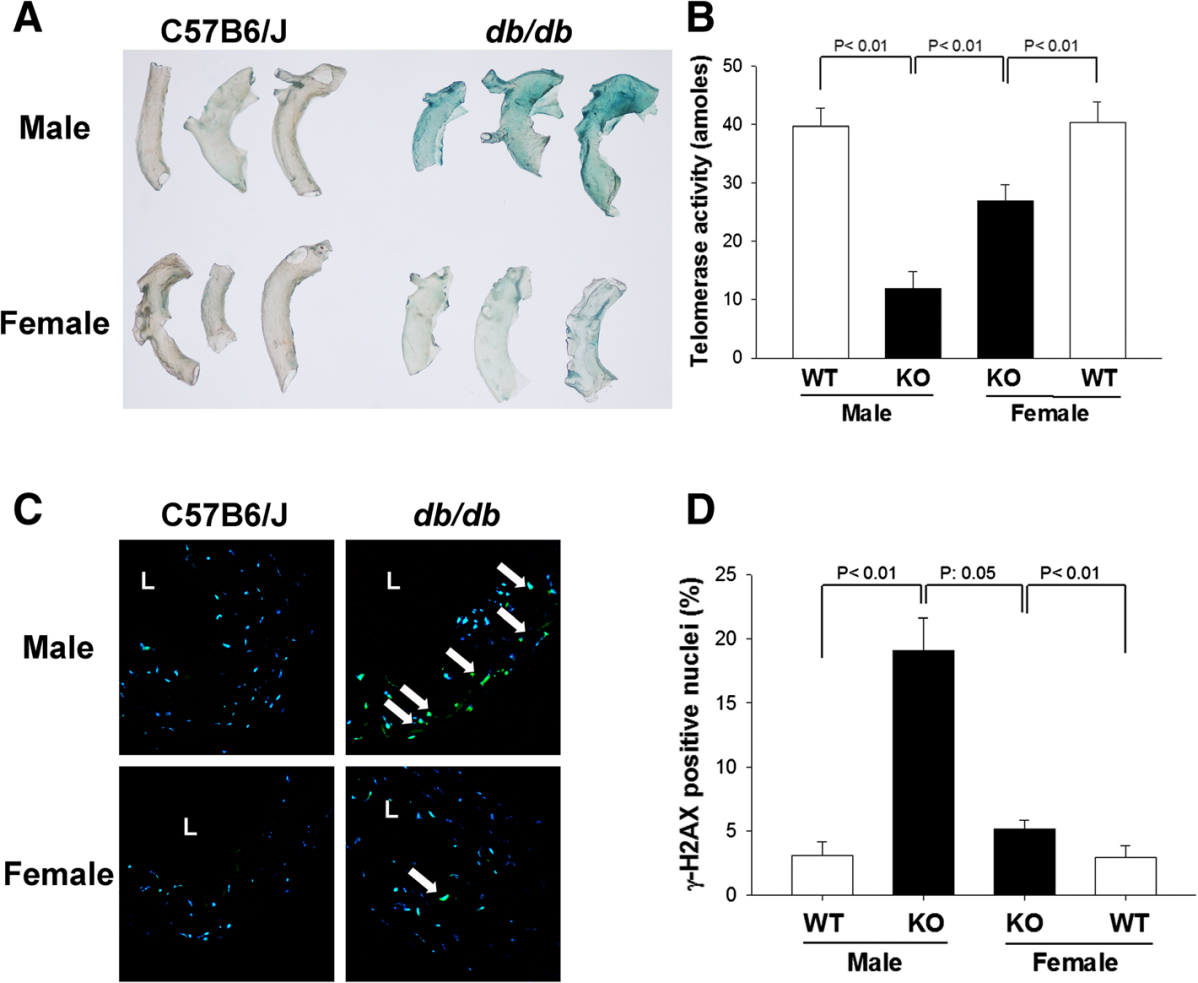
**Analysis of cellular senescence and DNA damage in the aortas of *db/db *and wild-type mice. A**, Representative SA-β-galactosidase staining of senescent cells (blue) in descending thoracic aortas from male and female *db/db* and wild-type mice. **B**, Telomerase activity in aortas from male and female *db/db* and wild-type mice. **C**, Representative γH2AX immunostaining (green) of aortic cross-sections from male and female *db/db* and wild-type mice. L, lumen. Arrows indicate positive staining. **D**, The percentage of γH2AX-positive nuclei was compared among groups (n = 8 per group).

To compare the extent of vascular senescence in male and female *db/db* and wild-type mice *in vivo*, aortas from these mice were fixed and stained with SA-β-gal. SA-β-gal activity, indicated by blue color, was strong in aortas of male *db/db* mice, weak in aortas of female *db/db* mice, and absent in aortas of male or female wild-type mice (Figure 3A). When we examined telomerase activity in aortas from these mice, we observed significantly lower telomerase activity in the aortas of *db/db* mice than in those of wild-type mice, for both genders (*P <* 0.01). Furthermore, the telomerase activity in the aortas of male *db/db* mice was significantly lower than that in the aortas of female *db/db* mice (*P <* 0.01) (Figure 3B). In addition, aortic cross-sections stained with anti-γH2AX, indicated by green color, showed marked DNA damage in the vascular cell nuclei of male *db/db* mice that was not observed in male or female wild-type mice (Figure 3C). Aortic cross-sections of female *db/db* mice also showed several positively stained nuclei, but the percentage was significantly lower than that in male *db/db* mice (Figure 3D).

### LDL from male *db/db* mice causes endothelial cell senescence that can be blocked by estrogens

**Figure 4:**
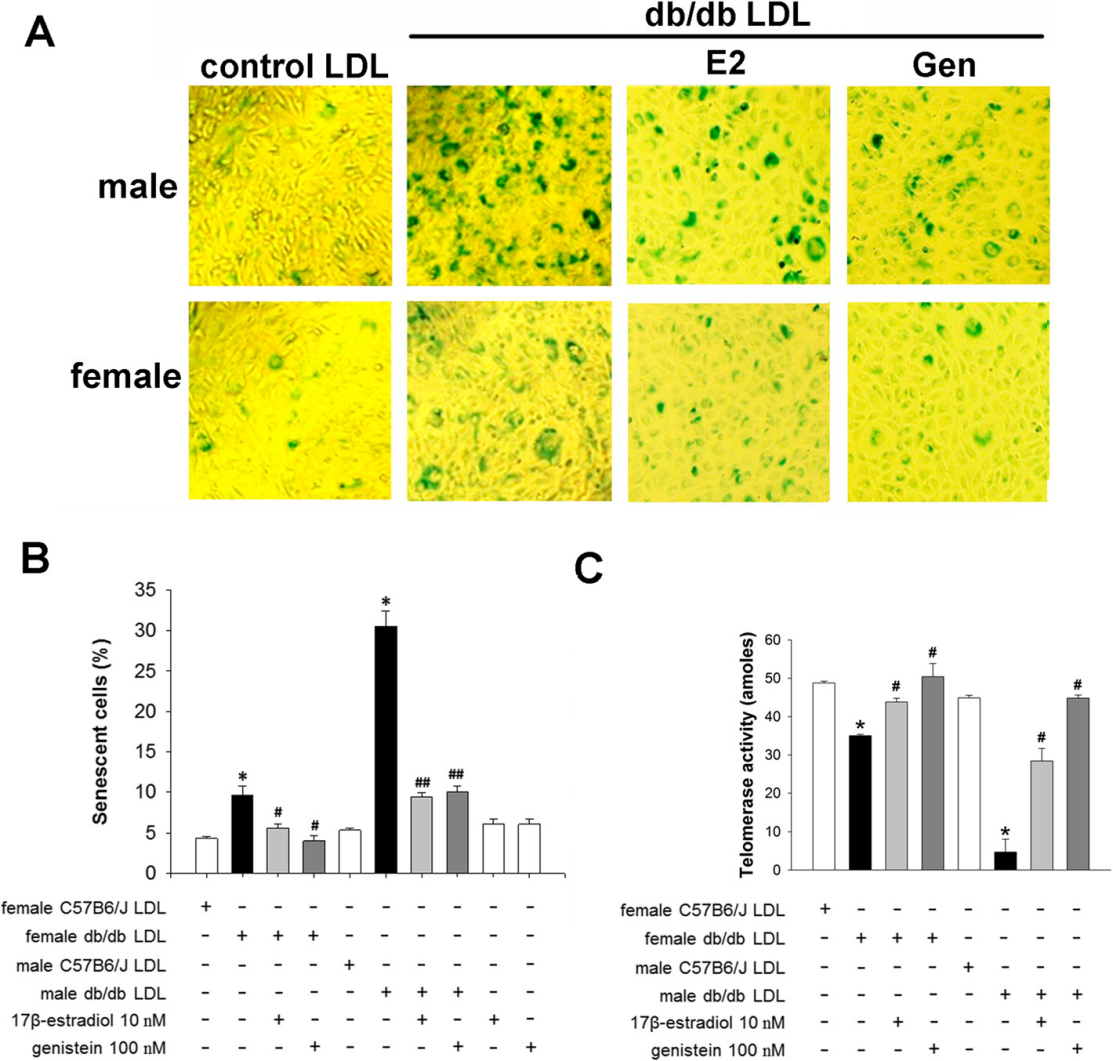
**Effect of LDL electronegativity and estrogens on bovine aortic endothelial cell (BAEC) senescence. A**, Representative images of SA-β-gal staining of senescent BAECs (blue) 5 days after incubation with 30 μg/mL LDL isolated from male or female *db/db* or wild-type mice in the presence or absence of pretreatment with 17β-estradiol (10 nM) or genistein (100 nM). **B**, The percentage of senescent cells and **(C)** telomerase activity in BAECs treated with LDL from male or female *db/db* or wild-type mice in the presence or absence of 17β-estradiol or genistein. n = 4 for each group. *P < 0.01 vs. gender-matched wild-type LDL, ^#^*P <* 0.05 and ^##^*P <* 0.01 vs. gender-matched *db/db* LDL.

To test our hypothesis that LDL with increased electronegativity induces vascular senescence, we exposed BAECs to LDL from male or female *db/db* or wild-type mice and analyzed SA-β-gal staining and telomerase activity in the treated cells. BAECs treated with LDL from male *db/db* mice had a significantly higher percentage of SA-β-gal–positive stained cells (*P <* 0.01) and a significantly lower amount of telomerase activity (*P <* 0.01) than did BAECs treated with LDL from male wild-type mice (Figure 4). In addition, BAECs treated with LDL from female *db/db* mice had a significantly higher percentage of SA-β-gal–positive stained cells (*P <* 0.01) and a significantly lower amount of telomerase activity (*P <* 0.01) than did BAECs treated with LDL from female wild-type mice, although the effects of LDL from female *db/db* mice were not as strong as those of LDL from male *db/db* mice (Figure 4). Furthermore, when we examined whether estrogens confer protection against LDL from *db/db* mice, we found that pretreatment of BAECs with 17β-estradiol (10 nM) or genistein (100 nM) significantly attenuated the percentage of SA-β-gal–stained cells and blocked the effects on telomerase activity induced by LDL from male or female *db/db* mice (*P <* 0.01 or *P <* 0.05) (Figure 4).

### Toxicity induced by LDL from male ***db/db*** mice is mediated by increased TNF-α and LOX-1 expression in endothelial cells

**Figure 5:**
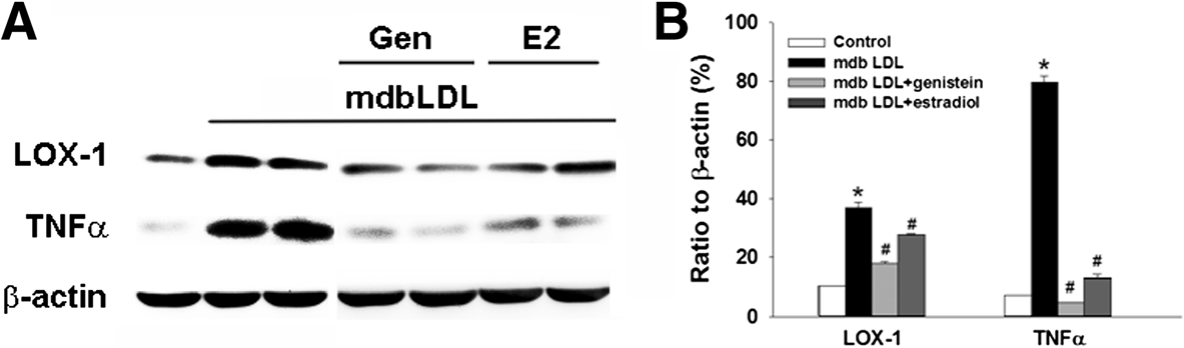
**LOX-1 and TNF-α expression in bovine aortic endothelial cells (BAECs) treated with LDL and/or estrogens. A**, Representative Western blot showing LOX-1 and TNF-α protein expression in BAECs treated with LDL from male *db/db* mice (mdbLDL) in the presence or absence of pretreatment with 100 nM genistein (Gen) or 10 nM 17β-estradiol (E2). **B**, Quantification of protein expression shown in **(A)**, expressed as a percent ratio to the expression of β-actin (internal loading control) (n = 4). *P < 0.05 vs control-treated BAECs, ^#^*P <* 0.05 vs. mdbLDL-treated BAECs.

To study the specific mechanisms underlying the effects of LDL and estrogen on vascular senescence, we examined LOX-1 and TNF-α expression in BAECs treated with LDL from male *db/db* mice in the presence or absence of pretreatment with 17β-estradiol or genistein (Figure 5A and B). In BAECs treated with LDL from male *db/db* mice, LOX-1 and TNF-α protein levels were significantly upregulated when compared with those in PBS-treated cells (*P <* 0.05). In addition, pretreatment with 17β-estradiol or genistein decreased LOX-1 and TNF-α protein expression induced by LDL from male *db/db* mice (*P <* 0.05 vs. LDL-treated cells; Figure 5A and B).

## Discussion

### Significance of increased LDL electronegativity in men with MetS compared to women with MetS

Cardiovascular disease differs between men and women. Among men and women aged 20 to 44, the prevalence of cardiovascular disease is one-third higher in men than in women (34% versus 24%, respectively) [29]. In a middle-aged group of MetS subjects, we found that increased plasma L5 levels in these subjects are more pronounced in men than in women, which may explain why men are at higher cardiovascular risk than are women and require early medical treatment, such as statin administration. In addition, in male mice with MetS, we observed increased LDL electronegativity compared to wild-type mice. We found in mice with MetS and in cultured BAECs that gender corresponded with the extent of vascular senescence. Interestingly, we observed a protective effect of 17β-estradiol or genistein against electronegative LDL–induced senescence. Our findings collectively suggest that gender disparities in LDL electronegativity may underlie the increased propensity to CAD in men.

### Potential contribution of increased ApoCIII content to the harmful effects of electronegative LDL

When fed a Western diet, *db/db* mice have an atherogenic lipoprotein profile characterized by increased levels of VLDL cholesterol and LDL-C [30]. Consistent with the findings of a previous study, we found that male and female *db/db* mice fed for 6 months with a standard chow diet showed evidence of weight gain, hyperglycemia, and hypercholesterolemia [31]. However, these traits did not differ between our male and female *db/db* mice and, thus, cannot explain the differences observed in vascular senescence in these animals.

We previously showed that plasma levels of L5 are higher in patients with diabetes mellitus than in healthy subjects [18] and that levels of the most electronegative subfraction of very-low-density lipoprotein, called V5, are higher in patients with MetS than in healthy subjects [32]. Recently, a clinical study, Prospective Urban Rural Epidemiology (PURE), of South Africans showed that some dietary fatty acids affect blood lipid profiles more unfavorably in men than in women [33]. To further characterize gender-influenced lipid abnormalities that occur in diabetes and MetS, we compared the electronegativity of LDL and the content of ApoCIII between male and female *db/db* mice and isogenic wild-type mice. LDL from male *db/db* mice was more electronegative than LDL from wild-type mice and contained more ApoCIII than did LDL from female *db/db* mice or wild-type mice. In addition, in each group of mice, ApoCIII content was greater in the LDL that induced more significant vascular senescence, indicating that ApoCIII may be one of the components of highly electronegative LDL responsible for its harmful effects. It has been well-established that ApoCIII is atherogenic and contributes to vascular senescence and coronary heart disease [34]. ApoCIII plays a pivotal role in regulating lipoprotein metabolism by inhibiting receptor-mediated uptake by the liver, which leads to the accumulation of triglycerides in ApoCIII-containing lipoproteins [35]. Other toxic components of LDL previously associated with MetS include oxidized LDL, small dense LDL, and other modified forms of LDL [36,37]. However, copper-oxidized LDL is artificially derived, and, although small dense LDL is a plausible concept, neither has yet to be isolated from the plasma for mechanistic scrutiny. ApoB has also been reported to be associated with MetS [38], [39]. In a previous study, we found that L5 contains fragmented ApoB100, which is most likely a consequence of longer LDL residence times in the circulation [40].

### Protective role of female hormones against the harmful effects of electronegative LDL

Among women, the cardiovascular death rates are 3 times higher after menopause than before menopause [41], indicating a protective role for female hormones that decline with age. In addition, the presence of type 2 diabetes aggravates cardiometabolic risk in postmenopausal women, as demonstrated by more atherogenic lipid and proinflammatory profiles [42]. In patients with type 1 diabetes, gender-related differences in cardiometabolic risk (ie., obesity and dyslipidemia) have also been observed, which may result from different outcomes after treatment between male and female patients [43]. In our *in vivo* and *in vitro* experiments, we found that female *db/db* aortas exhibited less cellular senescence than did male *db/db* aortas, and LDL isolated from female *db/db* mice caused less BAEC senescence than did LDL from male *db/db* mice. Furthermore, 17β-estradiol or genistein reduced *db/db* LDL–induced senescence. The differences observed between male and female *db/db* mice, combined with our evidence for a protective role of estrogens, indicate that female hormones may protect the arterial endothelium against senescence *in vivo,* either by altering the electronegativity of LDL or by inhibiting electronegative LDL receptor–mediated signaling pathways, such as the LOX-1 signaling pathway. In support of our findings, observational studies have shown that estrogen therapy may significantly decrease the risk of CAD by counteracting oxidized LDL–induced endothelial dysfunction [44] and by inhibiting endothelial progenitor cell senescence [45]. Other research has indicated that estrogen does not confer protection by affecting vascular relaxation [46] and that its protective effects may be independent of lipid levels [47]. Brunelli and colleagues [48,49] have shown that 17β-estradiol could bind to a single specific and saturable binding site of ApoB100 and prevent the misfolding of its structure on electronegative LDL without changing the electronegativity and phospholipase activity of LDL. Therefore, the modification of ApoB100 may play an important role in the underlying mechanisms by which estrogen provides protection, although this possibility needs further investigation.

### Regulation of TNF-α and LOX-1 expression by electronegative LDL and estrogen

Recently, genetic analyses performed in various animal models have shown that phosphoinositol-3-kinase (PI3K)/Akt, TNF-α, and other metabolic pathways are crucial for cellular senescence [1]. Thus, it is possible that highly electronegative LDL promotes endothelial senescence by inhibiting the PI3K/Akt pathway and by increasing TNF-α via LOX-1 [19]. In the present study, we found that LDL isolated from *db/db* mice enhanced both LOX-1 and TNF-α expression and that pretreatment of BAECs with 17β-estradiol and genistein significantly diminished these effects. Our findings correspond to those previously reported showing that estrogen decreases TNF-α expression and oxidized LDL–induced endothelial cell dysfunction [50].

## Conclusion

In conclusion, our data have suggested that, compared with wild-type mice, the more prominent DNA damage and endothelial senescence observed in male *db/db* mice may be partly attributed to more electronegative and, thus, more toxic LDL. Furthermore, estrogens may provide systemic hormonal protection against the harmful effects of electronegative LDL. Our findings indicate that gender disparities in LDL-induced cardiovascular damage may impact the development of CAD.

## Abbreviations

MetS: Metabolic syndrome; CAD: Coronary artery disease; HDL-C: High-density lipoprotein cholesterol; LDL-C: low-density lipoprotein cholesterol; ApoCIII: Apolipoprotein CIII; db/db: Homozygous leptin receptor deficient; SA-β-gal: Senescence-associated– β-galactosidase; Anti-γH2AX: Anti–phospho-histone H2AX; BAEC: Bovine aortic endothelial cell; LOX-1: Lectin-like oxidized LDL receptor-1; TNF-α: Tumor necrosis factor-α; PI3K: Phosphoinositol-3-kinase.

## Competing interests

The authors declare that they have no competing interests.

## Authors’ contributions

LAS performed experiments, literature reading, manuscript writing, and problem solving and supervised the clinical and bench portions of the study. CWY performed experiments and literature reading. CHC performed experiments and literature reading. HJF performed statistical analysis and acted as a consultant. SMY performed experiments and literature reading. CCM performed experiments. HB participated in the writing of the manuscript. SMJ performed literature reading and problem solving. CKC performed literature reading, problem solving, and study design and co-mentored the first authors. CCH directed the study and performed literature reading, manuscript writing and revising, and problem solving. All authors read and approved the final manuscript.
